# Quantization-Mitigation-Based Trajectory Control for Euler-Lagrange Systems with Unknown Actuator Dynamics

**DOI:** 10.3390/s20143974

**Published:** 2020-07-17

**Authors:** Yi Lyu, Qiyu Yang, Patrik Kolaric

**Affiliations:** 1 School of Computer, University of Electronic Science and Technology of China Zhongshan Institute, Zhongshan 528400, China; yi.lyu@foxmail.com; 2 School of Automation, Guangdong University of Technology, Guangdong Key Laboratory of IoT Information Technology, Guangzhou 510006, China; 3 UTA Research Institute, The University of Texas at Arlington, Fort Worth, TX 76118, USA; patrik.kolaric@mavs.uta.edu

**Keywords:** networked network, Euler-Lagrange systems, quantization mitigation, trajectory control

## Abstract

In this paper, we investigate a trajectory control problem for Euler-Lagrange systems with unknown quantization on the actuator channel. To address such a challenge, we proposed a quantization-mitigation-based trajectory control method, wherein adaptive control is employed to handle the time-varying input coefficients. We allow the quantized signal to pass through unknown actuator dynamics, which results in the coupled actuator dynamics for Euler-Lagrange systems. It is seen that our method is capable of driving the states of networked Euler-Lagrange systems to the desired ones via Lyapunov’s direct method. In addition, the effectiveness and advantage of our method are validated with a comparison to the existing controller.

## 1. Introduction

Recent decades have witnessed that the research on networked control systems is also one of the most important topics in the current academic and industrial field [1,2,3]. Different pieces of equipment or devices are connected through the network, provides more flexibility and resilience to different working conditions and environments. Please note that as the number of connected devices increases, or as the size of the networked system expands, it becomes a challenge as to how to use the limited computational resources for communicating [4].

From a perspective of the information flow, a problem of the limited computational resources can be regarded as a problem of the bandwidth limitations, wherein the transmitted signal is quantized through the networked systems. Thus, it leads to a challenge that how to control a quantized system. Along this line, some results of handling the quantization phenomena have been reported in the control community. Results on limited data rates were documented in [2]. The work of [3] addressed the communication constraint issue by placing the encoder and decoder in the control diagram. In contrast to the linear system in [5], works such as [6,7] extended the quantized feedback problem to the non-linear systems. Since the quantization was sector-bounded, Fu and Xie [8] changed the quantized feedback design into the robust control design. Considering that the input logarithmic quantizer may result in oscillation, Hayakawa et al. [9] gave a remedy using a hysteretic quantizer. It was reported in [10] that the input quantization problem can be handled using the backstepping-based design. Xing et al. [11] considered an output-feedback design problem for unknown nonlinear systems with the quantization of the system input. Xie et al. [12] proposed a neural-network-based asymptotic control algorithm to study unknown input quantization control problems for nonlinear systems using backstepping control design. Zhou et al. [13] extended [10] and showed that the Lipschitz condition was not necessary for the nonlinear functions with the quantization at the input.

Please note that Euler-Lagrange systems have significant advantages in modelling the dynamical processing for industrial applications, such as [14,15,16,17,18,19]. However, it is non-trivial to apply the linear system-based results to control the Euler-Lagrange systems. Difficulties include not only Euler-Lagrange systems themselves are nonlinear but also they might involve different kinds of nonlinearities [20,21]. Please note that actuators have been regarded as an essential unit in the control systems. When actuators are not working in perfectly linear phenomena, actuators nonlinearities [22] including deadzone, backlash, friction, and hysteresis would be imposed on the Euler-Lagrange systems. Therefore, how to model the nonlinearized actuator dynamics and how to tackle the problem of unknown actuator dynamics appear to be crucial problems that need further research. The work of [23] considered the motion control problem in linear resonant actuators by using an estimator for estimating the position and a motion controller. As documented in [24], the control problem of actuator nonlinearities was modelled for a class of nonlinear systems. Later, actuator failures, which can be regarded as a specific but difficult form of actuator nonlinearities, were investigated in [25]. Recently, Chen et al. [26] proposed a dynamic gain-based approach for multi-input and multi-output system with unknown input coefficients, which turned out to be related to the problem of actuator nonlinearities. The work of [27] studied the control problem of friction and hysteresis on the geared drives, and proposed a sensorless torsion control for elastic-joint robots. Results are obtained for multi-agent systems with external disturbances and unknown input nonlinearities in [28]. Lin et al. [29] considered the cooperative navigation control of mobile robots in an unknown environment using a neural fuzzy controller. The work of [30] considered unknown input Bouc-Wen hysteresis control problem and handled it using adaptive control. Although advances have been reported, the majority of the existing works are not designed for Euler-Lagrange systems with unknown quantization. This becomes a key question to investigate practical networked systems such as unmanned vehicle systems and teleoperated manipulators [31,32] with the capability of modelling the nonlinearities that happen in the communication networks [33].

In this paper, we aim to study a control problem of Euler-Lagrange systems with unknown actuator dynamics. In our case, the designed control signal is first endowed with the quantization to capture the limited bandwidth in the networked control system. After that, the quantized signal is allowed to pass through unknown actuator dynamics, which results in an unknown coupled dynamics control problem for Euler-Lagrange systems. We solve such a problem by proposing a quantization-mitigation-based adaptive control method. It is seen that our controller, together with its estimation laws, is capable of changing unknown time-varying input coefficients caused by the quantization and actuator dynamics into a problem of unknown constant input coefficients. Then, we solve the unknown constant input using an adaptive dynamic gain-based approach. Using our control scheme, the analysis shows that one estimation law is sufficient to handle unknown quantized actuator dynamics problem, regardless of the number of the actuator channels in the network. It is seen that our quantization-mitigation-based trajectory control works effectively for networked Euler-Lagrange systems both in the theoretic stability analysis and in the simulation case study. The proposed system model and its control result are important for the Euler-Lagrange systems that are operating in a networked workplace. In the networked control systems, the issue of the limited bandwidth has been studied and modelled by the quantization phenomenon [1]. Through our control design, we show that the tracking performance of the Euler-Lagrange systems is ensured even under the quantized actuator dynamics.

The organization of the remaining parts of this paper is briefly introduced as follows. In Section 2, we first model the networked Euler-Lagrange systems with unknown actuator dynamics. In Section 3, we review the structural property of Euler-Lagrange systems, propose an adaptive control-based method to handle unknown actuator dynamics and quantization for Euler-Lagrange systems, and proved its stability via Lyapunov’s direct method. In Section 4, the proposed method is tested through a case study, and its effectiveness is confirmed. In Section 5, a conclusion of this paper is attained.

## 2. Problem Formulation

In this section, we will consider a class of unknown Euler-Lagrange systems that contains unknown quantization on the input and unknown actuator dynamics. The dynamics for such Euler-Lagrange systems are modelled as the following nonlinear equation [34]:

(1) $$V\left(\chi \right)\ddot{\chi }+H\left(\chi ,\dot{\chi }\right)\dot{\chi }+W\left(\chi \right)=\tau ,$$

where $\chi \in R^{L\times 1}$ is a system state vector, $V\left(\chi \right)\in R^{L\times L}$ denotes a positive definite inertial matrix; $H\left(\chi ,\dot{\chi }\right)\in R^{L\times L}$ representees the Coriolis and centrifugal matrix of the *i*th robotic arm; $W\left(\chi \right)\in R^{L\times 1}$ denotes the gravitational force vector; and $\tau \in R^{L\times 1}$ means the actual actuator signal applied to the Euler-Lagrange systems and plays a role of driving the state variable χ of Euler-Lagrange systems (1) to follow a predetermined trajectory reference $\chi_{d}$. Here, the actuator dynamics are unknown to the designer. It is noted that the term τ in our case denotes a control or command signal received from the wireless networks. The mathematical model of the actual actuator dynamics τ is detailed as

(2) $$\begin{array}{cc} \tau & =G(\varrho (u(t),t))\\ \; & =[G_{1}\left(\varrho_{1}\left(u_{1}\left(t\right),t\right)\right),G_{2}\left(\varrho_{2}\left(u_{2}\left(t\right),t\right)\right),\ldots ,G_{L}\left(\varrho_{L}\left(u_{L}\left(t\right),t\right)\right)], \end{array}$$

where the function $G_{i}\left(\cdot \right)$ denotes unknown actuator dynamics driven by the signal (·) and $\varrho_{i}\left(u_{i}\left(t\right)\right)$ implies an unknown nonlinear function that changes the designed controller $u_{i}\left(t\right)$ to $\varrho_{i}\left(u_{i}\left(t\right)\right)$ caused by the wireless communications in the networked systems.

**Figure 1:**
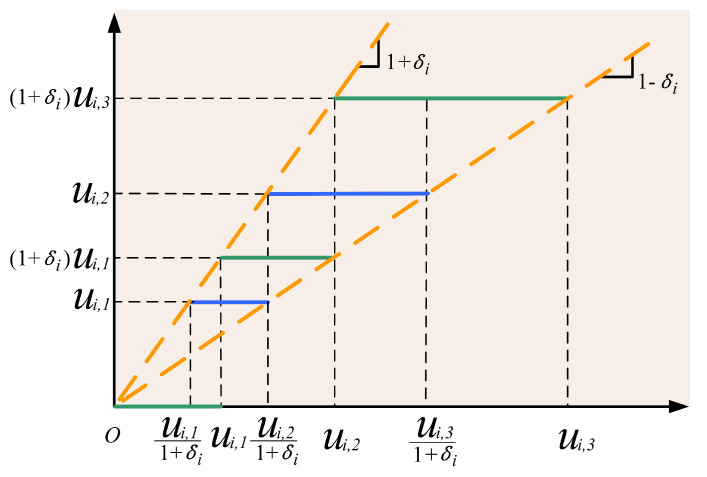
Quantization for networked communication.

Now, we model the dynamical procedure of the wireless communication. Although wireless communication provides a flexible way of controlling the system, the communication quality strongly relies on bandwidth. Under the limited bandwidth of wireless networks, we consider the designed actuator signal $u_{i}\left(t\right)$ in (2) subjected to the quantization [10], which can be regarded as a model to investigate practical systems such as telecommunicated vehicles or devices. For the simplification and convenience, we show the quantization with the input signal $u_{i}\left(t\right)$ and its output quantized signal $\varrho_{i}\left(u_{i}\left(t\right),t\right)$ in Figure 1, where it is clear that the quantizer $\varrho_{i}\left(u_{i}\left(t\right),t\right)$ is nonlinear and discontinuous severely twisting the designed signal $u_{i}\left(t\right)$.

The mathematical model of the quantization is expressed in a form as

(3) $$\varrho_{i}\left(u_{i}\left(t\right),t\right)=\{\begin{array}{c} u_{i}\left(t\right)sgn\left(u_{i}\left(t\right)\right),\\ \;\;\;\;\mathrm{if}\;\frac{u_{i}\left(t\right)}{1+\delta_{i}}<\left|u_{i}\left(t\right)\right|\leq u_{i},{\dot{u}}_{i}\left(t\right)<0,\\ \;\;\;\mathrm{or}\;u_{i}\left(t\right)<\left|u_{i}\left(t\right)\right|\leq \frac{u_{i}\left(t\right)}{1-\delta_{i}},{\dot{u}}_{i}\left(t\right)>0\\ u_{i}\left(t\right)\left(1+\delta_{i}\right)sgn\left(u_{i}\left(t\right)\right),\\ \;\;\;\;\mathrm{if}\;u_{i}\left(t\right)<\left|u_{i}\left(t\right)\right|\leq \frac{u_{i}\left(t\right)}{1-\delta_{i}},{\dot{u}}_{i}\left(t\right)<0,\\ \;\;\;\mathrm{or}\;\frac{u_{i}\left(t\right)}{1-\delta_{i}}<\left|u_{i}\left(t\right)\right|\leq \frac{u_{i}\left(1+\delta_{i}\right)}{1-\delta_{i}},\dot{u_{i}}\left(t\right)>0\\ 0,\;\;\mathrm{if}\;0\leq |u_{i}\left(t\right)|<\frac{u_{i,min}}{1+\delta_{i}},{\dot{u}}_{i}\left(t\right)<0,\\ \;\;\;\mathrm{or}\;\frac{u_{i,min}}{1+\delta_{i}}\leq \left|u_{i}\left(t\right)\right|\leq u_{i,min},{\dot{u}}_{i}\left(t\right)>0\\ \varrho_{i}\left(u_{i}\left(t_{-}\right)\right),\;\mathrm{if}\;other\;cases. \end{array}$$

where the symbol $\rho_{i}$ denotes the density of the quantized phenomena with $0<\rho_{i}<1$, $\delta_{i}=\frac{1-\rho_{i}}{1+\rho_{i}}$, $\varrho_{i}\left(u_{i}\left(t_{-}\right)\right)$ represents the quantized output, the value of which holds at the previous time, and $u_{i,j}\left(t\right)=\rho_{i}^{\left(1-j\right)}u_{i,\min}$ with $u_{i,\min}>0$ for j=1,2,…. To better capture the influence from the wireless communication, we assume that parameters in (3) are unknown to the controller design, which implies that the quantization is unknown.

Now, we give the modeling the actuator dynamics as

(4) $$G_{i}\left(v_{i}\right)=g_{i}^{v}\left(t\right)v_{i}+g_{i}^{u}\left(t\right),$$

where $v_{i}$ is a designation for a general input variable, and $g_{i}^{v}\left(t\right)$ denotes an unknown input coefficient with its sign being positive and $g_{i}^{u}\left(t\right)$ denotes an unknown bounded disturbance with its upper and lower bounds satisfying $0<{\underline{g}}_{i}^{u}\left(t\right)\leq \left|g_{i}^{u}\left(t\right)\right|\leq {\bar{g}}_{i}^{u}\left(t\right)$. For the control purpose, it is assumed that $g_{i}^{v}\left(t\right)$ has a lower bound that is strictly greater than the zero satisfying $g_{i}^{v}\geq {\underline{g}}_{i}^{v}>0$, which ensures that the controller signal is always effective in acting on the considered system. This assumption is standard and it follows from the literature such as [34].

**Remark** **1.** *The model in (4) can be employed to capture actuator nonlinearities including the deadzone, hysteresis, and backlash, which are frequently found in the practical systems and are important to the quality of the control systems. Take the deadzone nonlinearities for example. Let the input and output be v and dz(v) with the system dimension one. From the definition of deadzone phenomena, one has*

$$dz\left(v\right)=\{\begin{array}{ll} k_{r}\left(v-m_{r}\right), & ifv\geq m_{r}\\ k_{l}\left(v-m_{l}\right), & if\;v\leq m_{l}\\ 0, & if\;other\;cases \end{array},$$

*where $m_{r}$, $m_{l}$, $k_{l}$, and $k_{r}$ are bounded variables. Then, one changes the last row of deadzone model into the following form $k_{l}\left(v-m_{l}\right)-k_{l}\left(v-m_{l}\right)$, where $k_{l}\left(v-m_{l}\right)$ in the last row is bounded. Therefore, the deadzone phenomena can be reexpressed by the form of (4). This clarifies that (4) has the capability of modelling certain types of actuator nonlinearities.*

After combining the quantization (3) and the actuator dynamics (4), one has the coupled dynamics for the actuator of (1) as

(5) $$G_{i}(\varrho_{i}(u_{i}\left(t\right),t))=\{\begin{array}{c} g_{i}^{v}\left(t\right)u_{i}\left(t\right)sgn\left(u_{i}\left(t\right)\right)+g_{i}^{u}\left(t\right),\\ \;\;\;\;\mathrm{if}\frac{u_{i}\left(t\right)}{1+\delta_{i}}<\left|u_{i}\left(t\right)\right|\leq u_{i},{\dot{u}}_{i}\left(t\right)<0,\\ \;\;\;\;\mathrm{or}u_{i}\left(t\right)<\left|u_{i}\left(t\right)\right|\leq \frac{u_{i}\left(t\right)}{1-\delta_{i}},{\dot{u}}_{i}\left(t\right)>0\\ g_{i}^{v}\left(t\right)u_{i}\left(t\right)\left(1+\delta_{i}\right)sgn\left(u_{i}\left(t\right)\right)+g_{i}^{u}\left(t\right),\\ \;\;\;\;\mathrm{if}u_{i}\left(t\right)<\left|u_{i}\left(t\right)\right|\leq \frac{u_{i}\left(t\right)}{1-\delta_{i}},{\dot{u}}_{i}\left(t\right)<0,\\ \;\;\;\;\mathrm{or}\frac{u_{i}\left(t\right)}{1-\delta_{i}}<\left|u_{i}\left(t\right)\right|\leq \frac{u_{i}\left(1+\delta_{i}\right)}{1-\delta_{i}},\dot{u_{i}}\left(t\right)>0\\ g_{i}^{u}\left(t\right),\;\mathrm{if}\;0\leq \left|u_{i}\left(t\right)\right|<\frac{u_{i,min}}{1+\delta_{i}},{\dot{u}}_{i}\left(t\right)<0,\\ \;\;\;\;\mathrm{or}\frac{u_{i,min}}{1+\delta_{i}}\leq \left|u_{i}\left(t\right)\right|\leq u_{i,min},{\dot{u}}_{i}\left(t\right)>0\\ g_{i}^{v}\left(t\right)\varrho_{i}\left(u_{i}\left(t_{-}\right)\right)+g_{i}^{u}\left(t\right),\;\mathrm{if}\;other\;cases, \end{array}.$$

**Figure 2:**
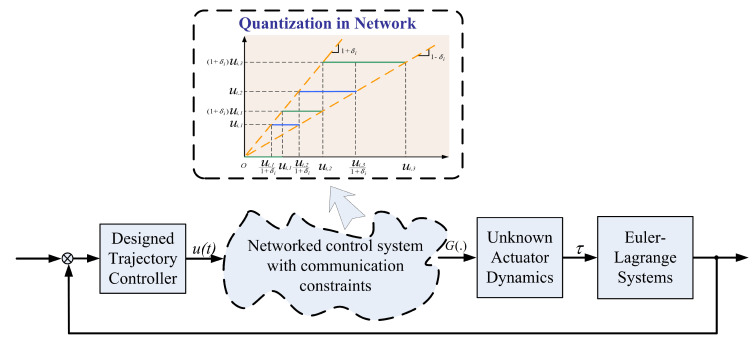
Networked Euler-Lagrange systems with unknown actuator dynamics.

Considering that the parameters including $g_{i}^{v}\left(t\right)$, $g_{i}^{u}\left(t\right)$, $\delta_{i}$ and $u_{i,min}$ in (5) are unknown to the designer, we call (5) unknown quantized actuator dynamics. It is noted that Equation (1), together with (5), is capable of modelling several equipment and devices communicating through networks including the teleoperated robot systems, unmanned vehicular systems, and sensor networks. The quantized actuator dynamics for networked Euler-Lagrange systems are shown in Figure 2.

We are ready to define the problem to be studied.

The Problem of Networked Euler-Lagrange Systems with Unknown Actuator Dynamics is to design a quantization-mitigation-based controller $u_{i}$ for the Euler-Lagrange systems (1) so that the controlled state χ converges to the predetermined state $\chi_{d}$, i.e., $\chi \left(t\right)\to \chi_{d}\left(t\right)$ and $\dot{\chi }\left(t\right)\to {\dot{\chi }}_{d}\left(t\right)$ as t→∞ under unknown quantized actuator dynamics (5).

## 3. Control Design for Networked Euler-Lagrange Systems with Unknown Actuator Dynamics

In this section, we will give our method to solve *the Problem of Networked Euler-Lagrange Systems with Unknown Actuator Dynamics* by the following three parts. To this end, we first review the structural properties of Euler-Lagrange systems. Then, a dynamic loop gain function-based control method is reviewed. Lastly, we give our main controller design and prove its stability analysis by using Lyapunov’s direct method.

### 3.1. Structural Properties of Euler-Lagrange Systems

In this subsection, we provide the structural properties of Euler-Lagrange systems as follows, which can also be found in the literature such as [34]:

**Property** **1.** *The matrix V(χ) in (1) is positive definite with $V^{T}\left(\chi \right)=V\left(\chi \right)>0$ satisfying*

(6) $$\xi_{min}\left(V\left(\chi \right)\right)\leq {∥V\left(\chi \right)∥}_{2}\leq \xi_{max}\left(V\left(\chi \right)\right),$$

*where $\left|\right|\cdot {\left|\right|}_{2}$ denotes a norm operator, and $\xi_{max}\left(V\left(\chi \right)\right)$ and $\xi_{max}\left(V\left(\chi \right)\right)$ are defined as the maximum and minimum eigenvalues of the matrix V(χ), respectively.*

**Property** **2.** *Considering $H(\chi ,\dot{\chi })$ and V(χ) in (1), one obtains that the matrix $2H\left(\chi ,\dot{\chi }\right)-\dot{V}\left(\chi \right)$ is skew-symmetric, where $\dot{V}\left(\chi \right)$ denotes the time derivative of V(χ). This implies that*

(7) $$x^{T}\left(2H\left(\chi ,\dot{\chi }\right)-\dot{V}\left(\chi \right)\right)x=0,\;\forall x.$$

**Property** **3.** *Given the variables χ, $\dot{\chi }$, α, and $\dot{\alpha }$ with the appropriate dimensions, the terms on the left side of (1) can be reexpressed as*

(8) $$V\left(\chi \right)\dot{\alpha }+H\left(\chi ,\dot{\chi }\right)\alpha +W\left(\chi \right)=ℵ\left(\chi ,\dot{\chi },\alpha ,\dot{\alpha }\right)\Omega ,$$

*with *Ω* denoting a column vector with unknown constant elements and $ℵ(\chi ,\dot{\chi },\alpha ,\dot{\alpha })$ being a known regression matrix.*

The above-mentioned three properties will be used for designing the quantization-mitigation- based controller.

### 3.2. Adaptive Method for Adjusting the Control Gain

As shown in the *Problem Formulation Section*, unknown quantized actuator dynamics (5) results in two parts, namely (1) input coefficients multiplying the controller and (2) unstructured disturbances. To address such two terms, we consider an adaptive method for adjusting the input coefficients and for tolerating the unstructured disturbances.

For the control purpose, we introduce a class of adaptive dynamic gains as [26]

(9) $$A(\phi )=\phi e^{\phi^{2}},$$

where ϕ is a real scalar. We summarize the adaptive dynamic gain-based result in the following lemma.

**Lemma** **1** ([26]). *Smooth functions U(t) and ϕ(t) are defined over the interval $\left[0,t_{d}\right)$ with U(t) nonnegative and ϕ(t) monotonic. Let ϕ(0) be bounded. The adaptive control gain A is given in (9). Then ϕ(t) and U(t) are bounded over $\left[0,t_{d}\right)$, if*

(10) $$\begin{array}{cc} U(t)\leq & -\int_{t_{0}}^{t}g_{\mu }A\left(\phi \left(\omega \right)\right)\dot{\phi }\left(\omega \right)\exp\left(\mu \omega -\mu t\right)d\omega \\ \; & +\int_{t_{0}}^{t}\dot{\phi }\left(\omega \right)\exp\left(\mu \omega -\mu t\right)d\omega +\gamma \left(t\right), \end{array}$$

*where γ(t) is an upper-bounded variable and μ and $g_{\mu }$ are positive constants.□*

### 3.3. Quantization-Mitigation-Based Trajectory Control Design

This section aims to provide a quantization-mitigation-based trajectory control design for Euler-Lagrange systems with unknown quantized actuator dynamics. It follows from adaptive control in [34] that the coordinate transformation is defined as

(11) $$\tilde{\chi }=\chi -\chi_{d},$$

(12) $${\dot{\chi }}_{r}={\dot{\chi }}_{d}-M_{r}\tilde{\chi },$$

(13) $$\varpi =\dot{\chi }-{\dot{\chi }}_{r},$$

where $M_{r}\in R^{L\times L}$ is set to be a positive definite matrix.

**Figure 3:**
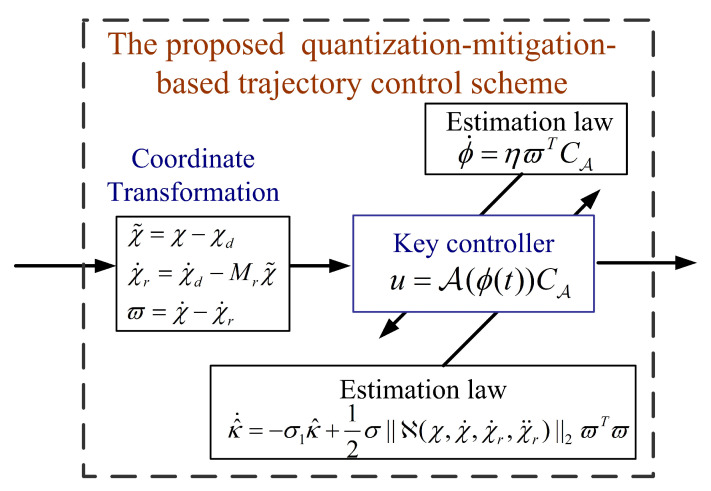
The proposed quantization-mitigation-based method.

Now, the quantization-mitigation-based controller for Euler-Lagrange systems with unknown quantized actuator dynamics is given as

(14) $$u=A\left(\phi \left(t\right)\right)C_{A},$$

with

(15) $$C_{A}=-\left(M_{e}+\frac{1}{2}I\right)e-\frac{1}{2}||ℵ\left(\chi ,\dot{\chi },{\dot{\chi }}_{r},{\ddot{\chi }}_{r}\right){\left|\right|}_{2}\hat{\kappa }\varpi ,$$

where $\hat{\kappa }$ denotes an estimation and its estimation law is specific later, the function of ℵ(·) is given in (8) by replacing variables $\chi ,\dot{\chi },\alpha ,\dot{\alpha }$ with $\chi ,\dot{\chi },{\dot{\chi }}_{r},{\ddot{\chi }}_{r}$, $M_{e}$ is a positive-definite matrix, and *I* denotes an identity matrix with an appropriate dimension. The estimation laws for (14) and (15) are designed as

(16) $$\dot{\phi }=\eta \varpi^{T}C_{A},$$

(17) $$\dot{\hat{\kappa }}=-\sigma_{1}\hat{\kappa }+\frac{1}{2}\sigma ||ℵ\left(\chi ,\dot{\chi },{\dot{\chi }}_{r},{\ddot{\chi }}_{r}\right){\left|\right|}_{2}\varpi^{T}\varpi ,$$

where η>0, σ>0, and $\sigma_{1}>0$ are design constants, κ(t) and ϕ(t) are initially chosen as κ(0)≥0 and ϕ(0)≥0. For better clarification, we depict the proposed quantization-mitigation-based trajectory control in Figure 3.

### 3.4. Stability Proof via Lyapunov’s Direct Method

In this subsection, we aim to give the main result of this paper and also present the stability analysis for the proposed quantization-mitigation-based controller.

The following theorem summarizes our main result.

**Theorem** **1.** *Let networked Euler-Lagrange systems be modelled as (1) with unknown quantized actuator dynamics (5), and the quantization-mitigation-based trajectory control be (14) with the estimation laws (16) and (17). Then, the controlled state variable χ, asymptotically, converges to the predetermined variable $\chi_{d}$, meanwhile, the state derivative $\dot{\chi }\left(t\right)$ also converges to ${\dot{\chi }}_{d}\left(t\right)$.*

**Proof.**  Under the coordinate transformations in (11)–(13), one substitutes (14) into (1) to obtain that

(18) $$H\left(\chi ,\dot{\chi }\right)\varpi +V\left(\chi \right)\dot{\varpi }=-ℵ\Omega +G\left(\varrho \left(u\left(t\right),t\right)\right)C_{A},$$

where *ℵ* and Ω are defined in (8) of *Property 3* with *ℵ* being a known regression matrix and Ω being an unknown constant vector.

To analyze the networked Euler-Lagrange systems with unknown quantized actuator dynamics, it follows the Lyapunov’s direct method that we define an auxiliary function as

(19) $$U\left(t\right)=\frac{1}{2}\varpi^{T}V\left(\chi \right)\varpi +\frac{1}{2}{\tilde{\kappa }}^{2}\sigma^{-1},$$

where ϖ is given in (13), V(χ) is a positive definite matrix satisfying (6) as shown in *Property 1*, and $\tilde{\kappa }$ is an estimation error defined as

(20) $$\tilde{\kappa }=\hat{\kappa }-\kappa ,$$

with $\hat{\kappa }$ being given in (17) and κ being specific later. Now, the derivative of (19) with respect to (18) is changed into

(21) $$\begin{array}{cc} \dot{U}\left(t\right)= & C_{A}+\varpi^{T}ℵ\Omega +\varpi^{T}[G\left(\varrho \left(u\left(t\right),t\right)\right)-C_{A}]+\dot{\hat{\kappa }}\tilde{\kappa }\sigma^{-1}\\ = & C_{A}+\dot{\hat{\kappa }}\tilde{\kappa }\sigma^{-1}+\varpi^{T}[G\left(\varrho \left(u\left(t\right),t\right)\right)-C_{A}]\\ \; & +\frac{1}{2}+\frac{1}{2}{\left||ℵ|\right|}_{2}\kappa \varpi^{T}\varpi , \end{array}$$

where $\kappa ={\left||\Omega |\right|}_{F}$. It is clear that κ is an unknown positive scalar given that Ω is unknown. Considering that the matrix $\dot{V}\left(\chi \right)-2H\left(\chi ,\dot{\chi }\right)$ is skew-symmetric in *Property 2*, one changes (21) into

(22) $$\begin{array}{cc} \dot{U}\left(t\right)\leq & -\varpi^{T}M_{e}\varpi +\varpi^{T}[G\left(\varrho \left(u\left(t\right),t\right)\right)-C_{A}]\\ \; & -\frac{1}{2}\varpi^{T}\varpi +\frac{1}{2}+\dot{\hat{\kappa }}\tilde{\kappa }\sigma^{-1}-\frac{1}{2}{\left||ℵ|\right|}_{2}\varpi^{T}\varpi \tilde{\kappa }\\ = & -\varpi^{T}M_{e}\varpi +\varpi^{T}[G\left(\varrho \left(u\left(t\right),t\right)\right)-C_{A}]\\ \; & -\frac{1}{2}\varpi^{T}\varpi -\sigma_{1}\hat{\kappa }\tilde{\kappa }\sigma^{-1}+\frac{1}{2}, \end{array}$$

where Young’s inequality is employed. Again, it follows from Young’s inequality that $-\hat{\kappa }\tilde{\kappa }$ in (22) satisfies

(23) $$\begin{array}{cc} -\hat{\kappa }\tilde{\kappa } & =-\tilde{\kappa }\left(\kappa +\tilde{\kappa }\right)\\ \; & \leq \frac{1}{2}\kappa^{2}-\frac{1}{2}{\tilde{\kappa }}^{2}, \end{array}$$

where both the result in (20) and Young’s inequality are used. From (5), it is clear that $\delta_{i}$ is a positive scalar. Therefore, it follows from (5) that

(24) $$g_{i}^{v}\left(t\right)u_{i}\left(t\right)<g_{i}^{v}\left(t\right)u_{i}\left(t\right)\left(1+\delta_{i}\right).$$

Moreover, $\varrho_{i}\left(u_{i}\left(t_{-}\right)\right)$ on the last row of (5) is bounded. It is thus define the maximum of $\varrho_{i}\left(u_{i}\left(t_{-}\right)\right)$, i=1,2,…,L as $g_{\max}$. Taking the similar technique for Deadzone analysis in *Remark 1*, the last two rows of (5) can be remodelled as forms like the first two rows. Substituting (16) and (23) into (22) yields

(25) $$\begin{array}{cc} \dot{U}\left(t\right)\leq & -\varpi^{T}M_{e}\varpi +\varpi^{T}[G\left(\varrho \left(u\left(t\right),t\right)\right)-C_{A}]\\ \; & -\frac{1}{2}\varpi^{T}\varpi +\frac{1}{2}-\sigma_{1}\sigma^{-1}\left(\frac{1}{2}{\tilde{\kappa }}^{2}-\frac{1}{2}\kappa^{2}\right)\\ \leq & -\varpi^{T}M_{e}\varpi -\sigma_{1}\sigma^{-1}\frac{1}{2}{\tilde{\kappa }}^{2}+\frac{1}{2}-\frac{1}{2}\varpi^{T}\varpi \\ \; & +\sigma_{1}\sigma^{-1}\frac{1}{2}\kappa^{2}+\varpi^{T}[-C_{A}+G\left(\varrho \left(u\left(t\right),t\right)\right)]\\ \leq & -\varpi^{T}M_{e}\varpi -\sigma_{1}\sigma^{-1}\frac{1}{2}{\tilde{\kappa }}^{2}+\frac{1}{2}g_{\max}\\ \; & +\varpi^{T}\left(A\left(\phi \left(t\right)\right)g_{\min}-1\right)C_{A}+\frac{1}{2}\\ \; & +\sigma_{1}\sigma^{-1}\frac{1}{2}\kappa^{2}. \end{array}$$

The result in (25) is further changed into

(26) $$\begin{array}{cc} \dot{U}\left(t\right)\leq & -\mu U\left(t\right)+\varpi^{T}\left(A\left(\phi \left(t\right)\right)g_{\min}-1\right)C_{A}+\gamma_{0}\\ \leq & -\mu U\left(t\right)+\left(A\left(\phi \left(t\right)\right)g_{\min}-1\right)\dot{\phi }\left(t\right)\frac{1}{\eta }+\gamma_{0}, \end{array}$$

where

(27) $$\mu =\min\{\frac{2\delta_{\min}\left\{M_{e}\right\}}{\delta_{\min}\left(V\left(\chi \right)\right)},\sigma_{1}\},$$

(28) $$\gamma_{0}=\frac{1}{2}g_{\max}+\sigma_{1}\sigma^{-1}\frac{1}{2}\kappa^{2}+\frac{1}{2},$$

(29) $$g_{\min}=\min_{i=1,2,\cdots ,L}\left(g_{i}^{v}\right).$$

**Remark** **2.** *We pause to highlight how to handle unknown quantized actuator dynamics as indicated in (26). Specifically, the time-varying input coefficients caused by the quantization and nonlinear actuator dynamics are multiplied with the adaptive gain A(ϕ(t)) in (9), the sign of which is always non-negative. This is ensured by the estimation law given in (16) with $C_{A}$ in (15). To this end, we are capable of handling the problem of time-varying input coefficients into a lower bounded input gain problem as $g_{\min}$ shown in (29). It will be seen that the designed estimation law plays a key role in achieving the asymptotic control for networked Euler-Lagrange systems under the quantized actuator dynamics.*

Now, we continue the proof of the proposed quantization-mitigation-based trajectory control design. Solving (26) with respect to the time over the interval [0,t] yields

(30) $$\begin{array}{cc} U(t)\leq & -V\left(0\right)\exp\left(-\mu t\right)+\frac{\mu }{\gamma_{0}}\\ \; & +\frac{1}{\eta }\int_{t_{0}}^{t}\dot{\phi }\left(\omega \right)\exp\left(\mu \omega -\mu t\right)d\omega \\ \; & -\frac{1}{\eta }\int_{t_{0}}^{t}g_{\min}A\left(\phi \left(\omega \right)\right)\dot{\phi }\left(\omega \right)\exp\left(\mu \omega -\mu t\right)d\omega \\ ≜ & \gamma +\frac{1}{\eta }\int_{t_{0}}^{t}\dot{\phi }\left(\omega \right)\exp\left(\mu \omega -\mu t\right)d\omega \\ \; & -\frac{1}{\eta }\int_{t_{0}}^{t}g_{\min}A\left(\phi \left(\omega \right)\right)\dot{\phi }\left(\omega \right)\exp\left(\mu \omega -\mu t\right)d\omega , \end{array}$$

where

(31) $$\gamma =-V\left(0\right)\exp\left(-\mu t\right)+\frac{\mu }{\gamma_{0}}.$$

Recalling the definition of U(t) in (19), it is reasonable to assume that V(0) is bounded. The boundedness of V(0), together with the boundedness of μ and $\gamma_{0}$, leads that γ on the right-hand side of (30) is bounded. It is clear that *Lemma 1* can be applied to (30) so that both U(t) and ϕ(t) are ensured bounded. This implies that all the signals in the closed-loop system are bounded after using the proposed quantization-mitigation-based trajectory control (14). The following analysis will show that the asymptotic control is also ensured even in the presence of unknown quantized actuator dynamics. Integrating the estimation law (16) over the time interval [0,t] together with the controller design (15) yields

(32) $$\begin{array}{cc} \phi (t)-\phi (0)= & \int_{0}^{t}\eta \varpi^{T}\left(\tau \right)C_{A}\left(\tau \right)d\tau \\ \geq & \int_{0}^{t}\eta \varpi^{T}\left(\tau \right)\varpi \left(\tau \right)d\tau . \end{array}$$

Now, we focus on the boundedness of the terms on the right-hand side of (32). From the above-mentioned analysis, one obtains that the signals ϕ(t), ϕ(0), and η are bounded. As an immediate result, the integral term $\int_{0}^{t}\eta \varpi^{T}\left(\tau \right)\varpi \left(\tau \right)d\tau$ on the right-hand side of (32) must exist and be finite. In addition, it is further obtained that the derivative of the signal ϖ is bounded. Subsequently, it follows from *Barbalat’s Lemma* that $\lim_{t\to \infty }\varpi^{T}\left(t\right)\varpi \left(t\right)=0$, which ensures that $\lim_{t\to \infty }\varpi \left(t\right)=0$.

Recalling the definition of ϖ(t) in (13), one obtains that $\dot{\chi }\left(t\right)\to {\dot{\chi }}_{d}\left(t\right)$ as t→∞. Now, recall the definitions of $\tilde{\chi }$ in (11) and $\dot{\chi }$ in (12), one rewrites ϖ(t) in (13) as

(33) $$\begin{array}{cc} \varpi (t)= & \dot{\chi }-{\dot{\chi }}_{d}+M_{r}\tilde{\chi }\\ = & \dot{\tilde{\chi }}+M_{r}\tilde{\chi }. \end{array}$$

Since ϖ(t) converges to zero and $M_{r}$ is a positive definite matrix defined in (12), then $\tilde{\chi }$ in (33) converges to zero so that $\chi \left(t\right)\to \chi_{d}\left(t\right)$ as t→∞. This completes the proof. □

**Remark** **3.** *In Theorem 1, it is proved that one estimation law is sufficient to handle the time-varying input coefficients caused by the quantized actuator dynamics, regardless of the number of the control channels in the network. The less estimation law is used in the control system, the more computational resources are saved for real-time performance. Therefore, the proposed quantization-mitigation-based result is important to the networked Euler-Lagrange systems from the perspective of the computation saving, especially for multiple devices sharing the common computational resources.*

## 4. Simulation and Experiment

In this scenario, we consider a Euler-Lagrange system as a robotic system, and control the robotic system through networks modelled by the quantization. The proposed quantization-mitigation-based controller in the previous section will be applied to the robotic system to test the system performance in the presence of unknown quantized actuator dynamics.

We follow the literature of [34] to give the dynamics of the robotic system as

(34) $$[\begin{array}{cc} V_{11} & V_{12}\\ V_{21} & V_{22} \end{array}][\begin{array}{c} {\ddot{\chi }}_{1}\\ {\ddot{\chi }}_{2} \end{array}]+[\begin{array}{c} Z_{11}\;Z_{12}\\ Z_{21}\;Z_{22} \end{array}][\begin{array}{c} {\dot{\chi }}_{1}\\ {\dot{\chi }}_{2} \end{array}]=[\begin{array}{c} \tau_{1}\\ \tau_{2} \end{array}],$$

where $V_{11}=\Omega_{1}+2\Omega_{3}\cos\left(\chi_{2}\right),$ $V_{12}=\Omega_{2}+\Omega_{3}\cos\left(\chi_{2}\right)+\Omega_{4}\sin\left(\chi_{2}\right)$, $V_{21}=V_{12}$, $V_{22}=\Omega_{2},$ $Z_{11}=-\Psi {\dot{\chi }}_{2}$, $Z_{12}=-\Psi \left({\dot{\chi }}_{1}+{\dot{\chi }}_{2}\right)$, $Z_{21}=\Psi {\dot{\chi }}_{1}$, $Z_{22}=0,$ and $\Psi =\Omega_{3}\sin\left(\chi_{2}\right)-\Omega_{4}\cos\left(\chi_{2}\right)$. Here, unknown constants $\Omega_{i}$ for i=1,2,3,4 are stacked into a vector as $\Omega ={\left[\Omega_{1},\Omega_{2},\Omega_{3},\Omega_{4}\right]}^{T}$. In this simulation, we let the designed controller first pass through the quantization (3). After that, the quantized signal passes through unknown actuator dynamics (4). This procedure leads that the actual input signal strictly follows (5) to set up the unknown quantized actuator dynamics problem for the Euler-Lagrange system. Considering that (34) is a multi-input and multi-output system, one designs the parameters of the quantization and the actuator dynamics in each control channel are the same as $\delta_{i}=0.8$ and $u_{i,\min}=0.1$ for i=1,2. Please note that the same parameters for each control channel are only for the simplification, and can also be set different using the proposed quantization-mitigation-based method. Initial states of the robotic system are randomly chosen. To implement our method, two estimation laws are built as required in (16) and (17) with the initials of such estimations being zeroes. That is, $\hat{\kappa }\left(0\right)=0$ and ϕ(0)=0. The desired trajectory is set to be $\chi_{d}={\left[0.5,0.2\right]}^{T}$.

**Figure 4:**
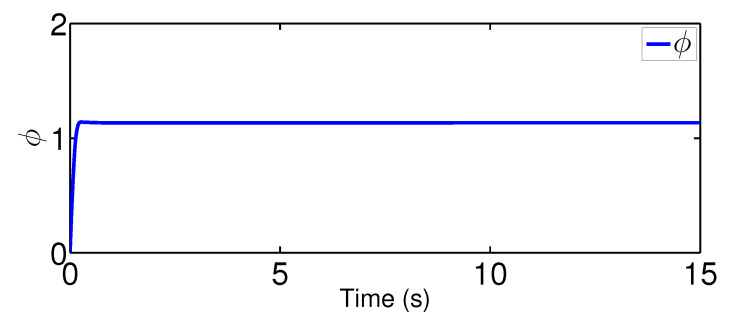
Trajectory of the estimation law ϕ.

**Figure 5:**
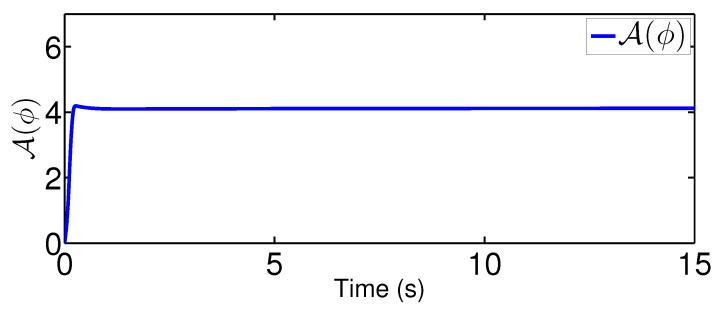
Adaptive dynamic gain A(ϕ).

**Figure 6:**
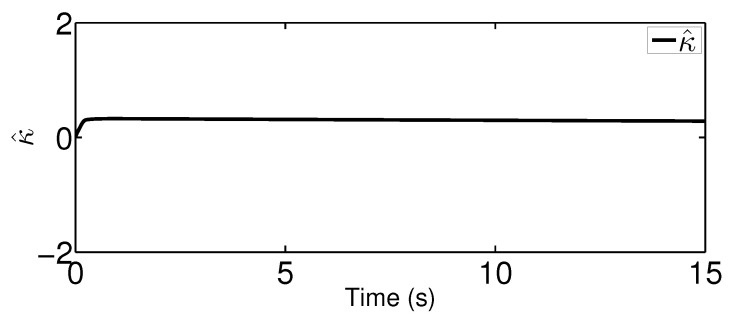
Trajectory of the estimation law $\hat{\kappa }$.

**Figure 7:**
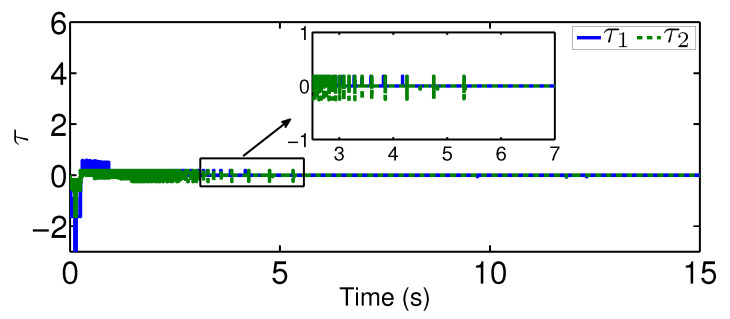
Control trajectory τ under the proposed controller.

**Figure 8:**
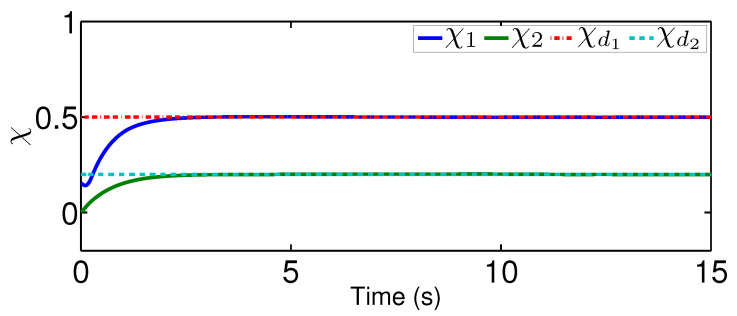
Performance χ under the proposed controller.

**Figure 9:**
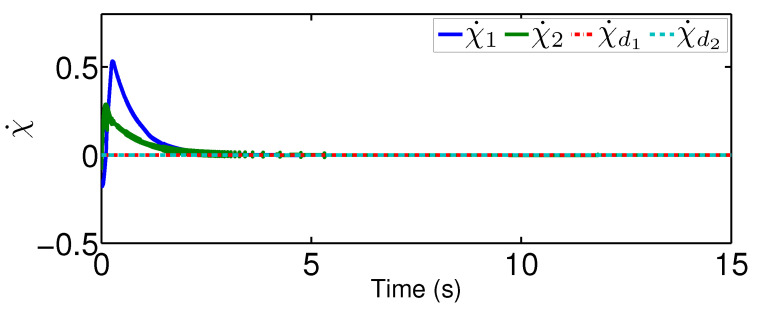
Performance χ under the proposed controller.

The results are plotted in Figure 4, Figure 5, Figure 6, Figure 7, Figure 8 and Figure 9, including the adaptive variables ϕ, A(ϕ), $\hat{\kappa }$, the control signal *u*, and trajectory performance χ and $\dot{\chi }$. In particular, the estimation law ϕ is given in Figure 4 and its adaptive dynamic gain A(ϕ) is given in Figure 5. It is seen that the estimation law for handling unknown quantized actuator dynamics reaches a steady value after the adaptation. The estimation law of $\hat{\kappa }$ for tunning the robotic parameters is presented in Figure 6. The actual actuator signal applied to the robot τ is plotted in Figure 7. It follows from Figure 4, Figure 5, Figure 6 and Figure 7 that the proposed quantization-mitigation-based trajectory controller is capable of driving the control and estimation laws in the Euler-Lagrange system to be bounded. As for the trajectory performance, we plot χ and $\dot{\chi }$, respectively, in Figure 8 and Figure 9, where the desired trajectories are also plotted for better clarification. From the results in Figure 8 and Figure 9, we conclude that the proposed quantization-mitigation-based trajectory controller works effectively under unknown quantized actuator dynamics.

**Figure 10:**
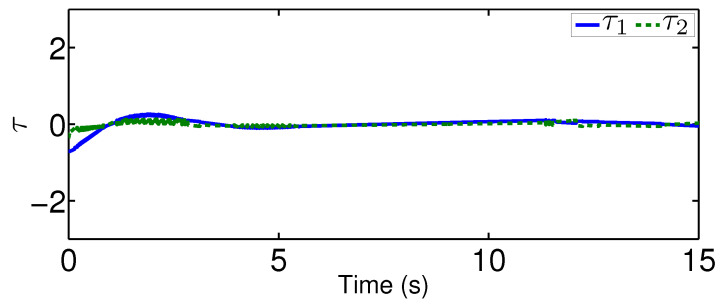
Control trajectory τ under the existing controller.

**Figure 11:**
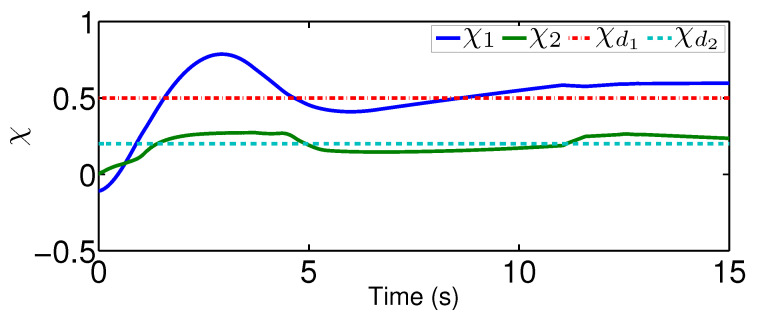
Performance χ under the existing controller.

**Figure 12:**
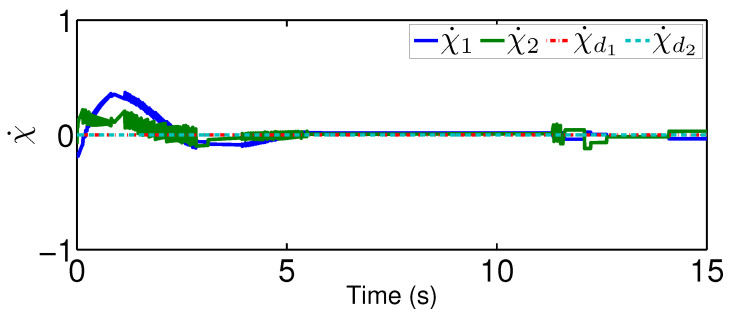
Performance $\dot{\chi }$ under the existing controller.

For the comparison, we test a fuzzy controller in [35], which uses the fuzzy logic systems to handle the system dynamics but does not contain an adaptive mechanism to reject unknown quantized actuator dynamics. Please note that we only change the controller from the proposed one to the fuzzy controller. The system dynamics, as well as the parameters for the quantization, are the same as that in the previous scenario. Under the existing fuzzy controller, the actual controller signal after the quantized actuator dynamics is plotted in Figure 10 and the actual trajectories of χ and $\dot{\chi }$ are depicted in Figure 11 and Figure 12. From Figure 11, there exists a steady error in controlling quantized actuator dynamics if the existing fuzzy controller is used. This confirms that the strong nonlinearities arising from the quantized actuator dynamics severely affect the system performance. Comparing the results in Figure 8 and Figure 9 and in Figure 11 and Figure 12, one concludes that the proposed quantization-mitigation-based method works better than the existing controller for the trajectory control of networked Euler-Lagrange systems.

To quantitatively describe the difference between the proposed controller and the existing controller, we define the index of the average error as follows

(35) $$I_{\tau_{i}}=\sqrt{\overset{N_{\tau_{i}}}{\underset{j=1}{\sum }}\frac{\tau_{i}^{2}\left(j\right)}{N_{\tau_{i}}}},$$

(36) $$I_{\chi_{1}}=\sqrt{\sum_{j=1}^{N_{\chi_{1}}}\frac{{\left(\chi_{1}\left(j\right)-0.5\right)}^{2}}{N_{\chi_{1}}}},$$

(37) $$I_{\chi_{2}}=\sqrt{\sum_{j=1}^{N_{\chi_{2}}}\frac{{\left(\chi_{2}\left(j\right)-0.2\right)}^{2}}{N_{\chi_{2}}}},$$

(38) $$I_{{\dot{\chi }}_{i}}=\sqrt{\sum_{j=1}^{N_{{\dot{\chi }}_{i}}}\frac{{\dot{\chi }}_{i}^{2}\left(j\right)}{N_{{\dot{\chi }}_{i}}}},$$

where $N_{\tau_{i}}$, $N_{\chi_{i}}$, and $N_{{\dot{\chi }}_{i}}$, for i=1,2, denote the total numbers of the signals $\tau_{i}$, $\chi_{i}$, and ${\dot{\chi }}_{i}$, respectively. The comparative results of $I_{\tau_{i}}$, $I_{\chi_{i}}$, and $I_{{\dot{\chi }}_{i}}$ are given in Table 1. It can be seen that the index of the control input $I_{\tau_{i}}$ under the proposed method is larger than that under the existing controller. This means that more control effort is used in the proposed method. However, the tracking performance $I_{\chi_{i}}$ and $I_{{\dot{\chi }}_{i}}$ under the proposed method is better than that under the existing controller.

## 5. Conclusions

In this paper, we investigated a trajectory control problem for wireless Euler-Lagrange systems with unknown actuator dynamics. We considered a control problem of trajectory control under the input quantization with unknown parameters. Subsequently, we derive the coupled dynamics that combine the quantization and actuator dynamics. To address such coupled dynamics, we proposed a quantization-mitigation-based trajectory control method, wherein adaptive control is employed to handle the time-varying input coefficients caused by the quantization nonlinearities. It was proved that the proposed method is capable of driving the states of networked Euler-Lagrange systems to the desired states, asymptotically. We tested our method and compare it with the conventional controller in the simulation and experiment section, wherein the effectiveness and advantage of our method are confirmed. In the realistic scenario, there are more complex dynamics in the networked control systems such as interference, packet delays, and unreliability. We will extend the result in this paper to consider the complex dynamics in the future work. The future research topic will include the learning-based control for unknown system dynamics such as [36,37].

## Figures and Tables

**Table 1:** Comparative results between the proposed method and the existing method [35].

	The Proposed Method	The Existing Method
$I_{\tau_{1}}$	0.28095	0.13352
$I_{\tau_{2}}$	0.08429	0.05116
$I_{\chi_{1}}$	0.06227	0.09195
$I_{\chi_{2}}$	0.03062	0.03881
$I_{{\dot{\chi }}_{1}}$	0.08463	0.09931
$I_{{\dot{\chi }}_{2}}$	0.04198	0.05410
